# Root canal pre-treatment and adhesive system affect bond strength durability of fiber posts ex vivo

**DOI:** 10.1007/s00784-021-03945-1

**Published:** 2021-06-14

**Authors:** Esra Kosan, Ana Prates-Soares, Uwe Blunck, Konrad Neumann, Kerstin Bitter

**Affiliations:** 1 Department of Oral Diagnostics and Digital Health and Health Services Research, Charité – Universitätsmedizin Berlin, corporate member of Freie Universität Berlin and Humboldt-Universität zu Berlin, Berlin, Germany; 2 Department of Operative and Preventive Dentistry, Charité – Universitätsmedizin Berlin, corporate member of Freie Universität Berlin und Humboldt-Universität zu Berlin, Berlin, Germany; 3 Institute of Biometry and Clinical Epidemiology, Charité – Universitätsmedizin Berlin, corporate member of Freie Universität Berlin and Humboldt-Universität zu Berlin, Berlin, Germany

**Keywords:** Self-etch adhesives, Root canal dentin, Ethanol pre-treatment, Push-out bond strength, Thermocycling

## Abstract

**Objectives:**

To investigate the effect of different pre-treatments on the long-term bond strength of fiberglass posts luted either with dual-curing self-etch adhesives and core build-up composites or with a self-adhesive resin (SAR) cement.

**Materials and methods:**

In total, 180 human root-filled teeth received post-space preparations and three different dentin pre-treatments (PTs): PT1, ethanol (99%); PT2, ethanol-tertiary-butanol-water-solution (AH Plus Cleaner, Dentsply Sirona; York, USA); and PT3, distilled water (control). Five luting systems were used: FU, Futurabond U (Voco; Cuxhaven, Germany); CL, Clearfil DC Bond (Kuraray Noritake; Okayama, Japan); GR, Gradia Core SE Bond (GC Europe NV; Leuven, Belgium); LU, LuxaBond Universal (DMG; Hamburg, Germany); and RX, RelyX Unicem 2 (3M; Minnesota, USA). Roots were cut into six slices (1 mm thick). From each root canal region, three slices were submitted to immediate and three to post-storage push-out testing. The latter were subjected to thermocycling (5–55°C, 6.000 cycles) and stored for six months in saline solution (0.9%, 37°C). Data were analysed using repeated measures ANOVA and chi-square tests (MV±SD).

**Results:**

Bond strength was significantly affected by material (*p*<0.0005), pre-treatment (*p*=0.016), and storage (*p*<0.0005; repeated-measures ANOVA). LU (18.8±8.1MPa) revealed significantly higher bond strength than RX (16.08±6.4MPa), GR (15.1±4.6MPa), CL (13.95±5.2MPa), and FU (13.7±6.3MPa). PT1 (16.5±6.9MPa) revealed significantly higher bond strength than PT3 (14.5±5.7MPa).

**Conclusions:**

A universal adhesive in self-etch mode combined with a core build-up material revealed higher bond strength than a SAR cement, both interacted positively with Ethanol pre-treatment.

**Clinical relevance statement:**

Ethanol (99%) rinsing can be recommended as part of post and core pre-treatment for the investigated luting systems.

## Introduction

The latest research highlighted the strong effect of a sufficient coronal restoration on the long-term survival of root canal–treated teeth [1]. In cases of high coronal substance loss, root canal posts are indicated to provide retention for the coronal restoration; and clinical research [2] suggests that teeth with extended tissue loss (no coronal walls above 2-mm gingival level) benefit from post placement. Concerning the selection of post material, a recent meta-analysis by Wang et al. indicates that fiber posts demonstrate a significantly higher mid-term survival rate than metal posts [3]. From a clinical perspective, the debonding of fiber-reinforced root canal posts was the most common mode of failure [4]. A finite element analysis (FEA) revealed that debonding of an adhesively luted fiber post transfers higher amounts of stress onto the tooth than a metal post [5]. Consequently, successful restoration of root canal–treated teeth using fiber posts is strongly dependent on a stable adhesive bond to root canal dentin [6]. This is often compromised due to irregular morphology of root canal dentin [8], limited visibility into the canal and lower numbers of dentinal tubules, complicating the adhesive luting procedure [9]. In addition,  moisture control of the root canal dentine after post-space preparation and etching is extremely difficult to achieve [10]. Consequently, protocols for adhesive luting to root dentin have been adapted according to the “ethanol wet bonding” strategy that was introduced as a pre-treatment for etch-and-rinse (ER) adhesives in order to facilitate the evaporation of residual water on coronal dentin and to stabilize the etched collagen matrix for resin infiltration [11]. For root canal, dentin rinsing with 100% ethanol for 60 s was suggested [9], aiming to simplify the procedure for clinical operations. Final ethanol rinsing resulted in higher immediate bond strength and lower nanoleakage in root canals [12]. However, there are conflicting results regarding the effects, which seem to strongly depend on the type of adhesive system applied [9, 13]. A meta-analysis by Sarkis-Onofre et al. [14] found evidence that simplified bonding systems like self-adhesive resin (SAR) cements revealed increased bond strength inside the root canal, since they are less technique-sensitive and easier to handle as they do not require etching or adhesive steps [15]. They are less susceptible to hydrolytic degradation than self-etch (SE) or ER systems according to a review by Ferracane et al. [15]. Nonetheless, SAR cements proved to be less suitable as core build-up materials, due to their insufficient marginal integrity [16]. Consequently, post-and-core systems have clinical advantages because post luting and core build-up can be performed in one step.

The latest approach to adhesive luting is the so-called universal or multimode adhesives. Manufacturers of universal adhesives often advertise them as applicable in total-etching, self-etching, or selective-etching mode [17] [18]. The performance of universal adhesives inside the root canal has only been investigated recently. So far, researchers have explored the effects of dentin moisture and application mode [19] on the bond strength of different universal adhesives inside the root canal. Studies comparing universal adhesives with other established luting agents are rare [20]. Therefore, the present study aims to compare the long-term bond strength of fiber posts luted with simplified dual-curing SE adhesives (part of which declared as “universal adhesives”) and a SAR cement while investigating the effect of three different pre-treatments on their bond strength to the canal walls. Additionally, the dentin–adhesive interface of each luting system was visualized and analysed to compare the hybrid layer formation. The null hypotheses of the present study were threefold: (1) pre-treatment does not affect bond strength, (2) there is no difference in bond strength among the tested materials, (3) storage and location do not affect bond strength inside the root canal.

## Materials and methods

A total of 180 caries-free human anterior teeth were obtained with written informed consent under an ethics-approved protocol (EA4/102/14) by the Ethical Review Committee of the Charité - Universitätsmedizin Berlin, Germany, and stored in 0.5% chloramine T solution for a maximum of 1 year after extraction until use. The minimum root length of selected teeth was 16 mm. Teeth were decoronated and received root canal treatment at working length 1 mm before the anatomical apex after initial scouting of the canal with a Flexicut file of ISO 10–20 and marked with a silicone stopper. A reciprocating file system (Wave one Gold, Dentsply Sirona; York, USA) was used for preparation until size 45.05. After every change of file, irrigation was performed with 2.5 ml NaOCl 1% solution. For root filling, standardized master points (Wave one Gold, size 45.05, Dentsply Sirona; York, USA) were used with warm vertical compaction technique (Calamus Dual, Dentsply Sirona; York, USA) and AH plus sealer (Dentsply Sirona; York, USA).

**Fig. 1:**
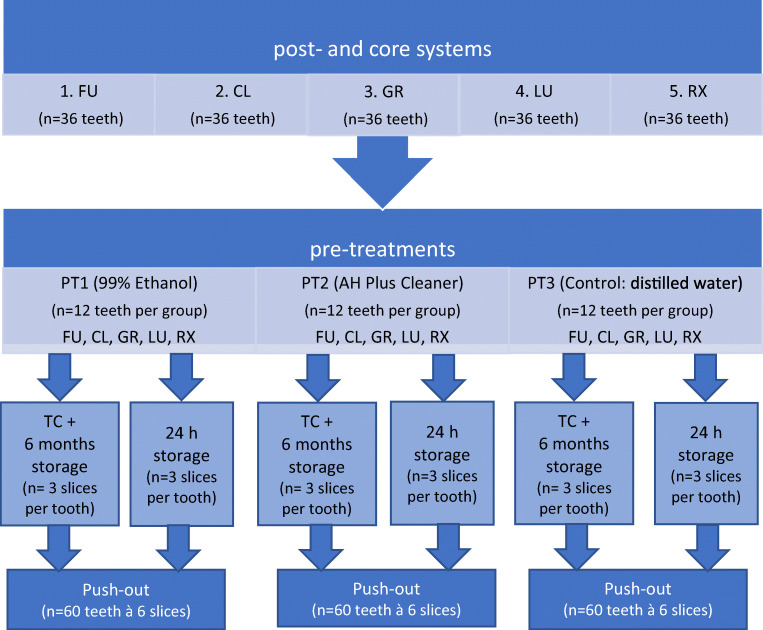
Flowchart of the experimental design

The teeth were divided into groups according to the flowchart displayed in Fig. 1. Post space preparation was performed with slow-speed drills provided by the manufacturers of the fiber post system. The depth of post-space preparation was 10 mm leaving 6 mm of gutta-percha as an apical sealing. In group 3, the post was luted at a depth of 12 mm, because of apical retentions which were excluded from the push-out test. Following post-space preparation, root canals were checked for remnants of gutta-percha and sealer using a stereomicroscope (Wild M5A, Wild Heerbrugg, Gais, Switzerland). After ensuring the cleanliness of each root canal, three different pre-treatments (PTs) were applied (Fig. 1).

For all groups, 5 ml of 1 % NaOCl (Aug. Hedinger GmbH & Co. KG; Stuttgart, Germany) was applied and ultrasonically activated twice, after 2.5 ml of the irrigant, according to the intermittent flush technique. This was performed with an ultrasonically oscillating file (Irri S File, VDW; Munich, Germany) for 2 × 30 s at 25–30 kHz using passive ultrasonic irrigation. In PT1, the canal was rinsed with 2.5 ml distilled water followed by rinsing with 2.5 ml 99% ethanol for 60 s (Ethanol Absolute, 99,9%, J.T. Baker, Avantor Performance Materials; Deventer, the Netherlands). In PT2, 2.5 ml distilled water was applied into the canal and subsequently dried using paper points followed by an ethanol-tertiary butanol-water solution (AH Plus Cleaner, Dentsply Sirona; York, USA), which was applied twice with a fully sucked microbrush for 30 s each. Afterwards, the canal was again rinsed with distilled water (2.5 ml), following the manufacturer’s instruction. In control group PT3, the root canal was solely rinsed with 5ml distilled water.

After drying every root canal with paper points, post and core systems were applied according to the manufacturer’s instructions (Table 1). In two of the five groups, universal adhesives in dual-curing mode were used: Futurabond U (FU) (Voco; Cuxhaven, Germany) and LuxaBond Universal (LU) (DMG; Hamburg, Germany). Clearfil DC Bond (CL) (Kuraray Noritake Dental Inc.; Tokyo, Japan) and Gradia Core SE Bond (GR) (GC Europe NV; Leuven, Belgium) are self-etch, dual-curing adhesives. RelyX Unicem 2 (RX) (3M; Minnesota, USA), a self-adhesive resin (SAR) cement, was used as a control. Except for Futurabond U, all adhesives were light-cured according to Table 1 using a Valo broadband LED light (WL: 395–480nm, 1.00/1.400–3.200 mW/cm, Ultradent Dental Medizinische Geräte GmbH & Co. KG; Brunnethal, Germany).

**Table 1:** All post-and-core systems used in the present investigation and the application mode of each system

Adhesive (LOT#)	Luting agent (LOT#)	Post (LOT#)	Manufacturers	Preparation	Application	
				Post	Adhesive	Luting agent
Futurabond U (1601051)	Rebilda DC (1605499)	Rebilda Post yellow (1549361) (cor. diam.: 2.0 mm, ap. diam.: 1.0 mm)	Voco; Cuxhaven, Germany	Clean post surface using 99% Ethanol; apply Ceramic Bond (#1439468) for 60 s; air dry	Activate the blister and mix with applicator tip; rub into root canal dentine for 20 s and air dry for 5 s; remove excess with paper points *NO LIGHT CURING!*	Apply composite using application tip provided by the manufacturer; During application: position application tip at the bottom of post space cavity and move it slowly from apical to coronal; insert post into canal and remove access; light cure for 40 s
Clearfil DC Bond A (39007) Clearfil DC Bond B (94005)	Clearfil DC Core Plus (AG0175)	Panavia Post No. 6 (5K004) (cor. diam.:1.64mm, ap. diam.: 0.8 mm)	Kuraray Noritake Dental Inc.; Okayama, Japan	Clean post surface using 99% Ethanol; apply K-Etchant Gel (#940018) for 5 s; rinse with water; apply Clearfil Ceramic Primer Plus (# AX0039) on surface; air dry	Mix liquid A and B using application tip; Rub into root canal dentine for 20 s and air dry for 5 s; remove excess with paper points; light cure for 20 s	Apply composite using application tip provided by the manufacturer; During application: position application tip at the bottom of post space cavity and move it slowly from apical to coronal; insert post into canal and remove access; light cure for 20 s
Gradia Core SE Bond A (1601081) Gradia Core SE Bond B (1601191)	Gradia Core (1611151)	GC Fiber Post blue (1512154) (cor. diam.: 1.6 mm, ap. diam.: 0.7 mm)	GC Europe NV; Leuven, Belgium	Clean post surface using 99% Ethanol; apply Ceramic Primer II (#1808061) on surface; air dry	Mix liquid A and B using application tip; *Rub into root canal dentine and leave for 30 s*; air dry for 10 s; remove excess with paper points; light cure for 10 s	Apply composite using application tip and *application gun* provided by the manufacturer; During application: position application tip at the bottom of post space cavity and move it slowly from apical to coronal; insert post into canal and remove access; light cure for 10 s
LuxaBond Universal A and B (747721)	LuxaCore Z Dual (747682)	LuxaPost green (752745) (cor. diam.: 1.5 mm, ap.diam.:0.9 mm)	DMG; Hamburg, Germany	Clean post surface using 99% Ethanol; the post surface is pre-silanized	Mix liquid A and B using application tip; Rub into root canal dentine for 20 s and air dry for 10 s; remove excess with paper points; repeat step 2 -4; light cure for 20 s	Apply composite using application tip provided by the manufacturer; During application: position application tip at the bottom of post space cavity and move it slowly from apical to coronal; insert post into canal and remove access; light cure for 20 s
RelyX Unicem 2 (658710)		RelyX Fiber-post size 2 (342291702) (cor. diam.: 1.6 mm, ap. diam.: 0.8 mm)	3M; Minnesota, USA	Clean post surface using 99% Ethanol; microporous post surface for resin retention	Apply self-adhesive resin (SAR) cement using application tip provided by the manufacturer; During application: position application tip at the bottom of post space cavity and move it slowly from apical to coronal; insert post into canal and remove access; light cure for 40 s	

After 24 h storage, roots were embedded in cold polymerising resin (Technovit 4071 “the fast,” Heraeus Kulzer, Wehrheim, Germany) and cut into six slices (thickness: 1.0 mm) perpendicular to the long axis of the root, starting from the coronal part of the root. This procedure resulted in two slices from the coronal post space, two slices from the middle post space, and two slices from the apical post space. The coronal and apical radiuses of the post in each slice were measured at a ×100–150 magnification using a digital multi-focus stereo microscope (Keyence Digitalmikroskop VHX - 500, Keyence Corporation, Keyence Deutschland GmbH; Neu-Isenburg, Germany). Occasionally entrapped voids were also documented, and slices were categorized into three groups: (1) void-free, (2) smaller voids (<0.2 mm diameter), and (3) numerous or larger voids (>0.2 mm diameter). From each root one slice each from the coronal, the middle and the apical regions were subjected to thermocycling (TC) and storage before push-out, while bond strengths of the remaining three slices were tested immediately. TC included 6.000 cycles between 5.0 and 55.0°C in deionised water, for 30 s each, using a thermocycler (self-construction). Subsequently, the samples were stored in isotonic saline solution (NaCl 0,9%, B. Braun Melsungen AG; Melsungen, Germany) for 6 months at 37°C which was changed weekly.

Push-out tests were performed with a universal testing machine (Zwick Roell Z010, Zwick GmbH & Co. KG; Ulm, Germany). The load was applied in apical-coronal direction at a crosshead speed of 0.5 mm min^-1^ using a 2.5 kN load cell. The load at debonding (Fmax) was recorded and bond strength was calculated (MPa) for Fmax (N) per bonded area (A [in square millimetre]). Area A was calculated using the formula *A*=π(*R*_1_+*R*_2_)√(*R*_1_−*R*_2_)^2^+h^2^ with *R*1 for the coronal and *R*2 for the apical radius of the post segment, while *h* was the thickness of each slice [21]. Afterwards, the fracture patterns of each sample were classified with the Keyence digital microscope VHX - 500 (with ×100–150 magnification) as follows: (1) cohesive failure of the post; (2) adhesive failure between root dentin and luting material; (3) adhesive failure between luting material and post; (4) mixed failure [2].

Additionally to the 180 teeth, two representative samples for each luting system were analysed using confocal laser scanning microscopy (CLSM) to visualize the adhesive interface of the different post- and core systems after the respective pre-treatment procedures. Therefore, samples were prepared as follows: The adhesives were mixed with 1% fluoresceine isothiocyanate (FITC, Merck KGaA; Darmstadt, Germany) and the luting materials were mixed with 1% rhodamine B isothiocyanate (RITC, Merck KGaA). Afterwards, the materials were applied according to Table 1 and cut horizontally into four 2-mm-thick discs, so that each area of the root canal was visualized. The discs were glued onto slides and polished with silicon carbide papers up to 4.000 grit using a polishing machine (Exakt Mikroschleifsystem, Exakt; Norderstedt, Germany). Subsequently, samples were analysed using the LSM 700 (Carl Zeiss Microscopy GmbH; Jena, Germany) in dual fluorescence mode using a ×50 objective and ×0.8 electronic zoom. Scanning of both dyes was performed with FITC (excitation: 488; emission: 516) and RITC (excitation: 555; emission: 580). The image size was 160 ×160 μm^2^ with a resolution of 1.024 × 1.024 pixels.

Furthermore, two additional intact samples from each group were scanned with a laboratory μCT (SKYSCAN 1275, Bruker; Billerica, USA) at 8-μm resolution and 55-ms exposure time in order to detect voids inside the luting material. Reconstruction was performed using NRecon 1.7 (Bruker; Billerica, USA). The samples were 3D rendered using CTVox software (Bruker; Billerica, USA) and analyzed qualitatively by one single operator.

Statistical analysis was performed using IBM SPSS statistics (IBM Corp. Released 2017. IBM SPSS Statistics for Windows, Version 25.0. Armonk, NY: IBM Corp.). Group means were compared using one-way ANOVA (UNI-ANOVA) and repeated measures ANOVA with Tukey’s post hoc tests. For metrical variables, means and standard deviations were reported (mean (SD)). For categorical variables, frequencies and percentages were calculated. Failure modes and void occurrences were analyzed using crosstabs and Pearson’s Chi-square test. The two-sided level of significance was *α*=0.05.

## Results

Mean push-out bond strengths were significantly affected by pre-treatment (*p*=0.016), storage (*p*<0.0005), and luting material (*p<*0.0005), but not by location (*p*=0.185; repeated measures ANOVA). Interactions between pre-treatment and luting material (*p*=0.011), storage and luting material (*p*<0.0005), and location and luting material (*p*<0.0005; repeated measures ANOVA) could be observed.

Overall, PT1 revealed significantly higher mean bond strength (16.5±6.9 MPa) than control group PT3 (14.5±5.7 MPa) (*p*<0.05; Tukey). PT2 (15.5±6.67 MPa) revealed no significant differences in mean bond strength compared to PT1 (*p*=0.332) and PT3 (*p*=0.293; Tukey). Initially tested samples (16.4±6.6 MPa) had an overall significantly higher mean bond strength compared to samples tested after TC and storage (14.6 ±6.2 MPa). Including the data before and after storage, samples of group LU (18.8±8.1 MPa) revealed significantly higher mean bond strength than those of RX (16.08±6.4 MPa), GR (15.1±4.6 MPa), CL (13.95±5.2 MPa), and FU (13.7±6.3 MPa; repeated measures ANOVA), which did not differ significantly from each other.

Regarding the interaction between luting material and pre-treatment, a stratified analysis revealed that CL (*p*=0.043), LU (*p*<0.0005), and RX (*p*<0.005; stratified UNI-ANOVA 1) were significantly affected by pre-treatment (Table 3). In group CL, PT1 revealed significantly higher mean bond strength compared to PT2 (*p*<0.05). In group LU, PT1 (*p*<0.0005) and PT2 (*p*<0.005) revealed significantly higher mean bond strength than PT3. In group RX, PT1 revealed significantly higher mean bond strength than PT3 (*p*<0.005; stratified UNI-ANOVA 1).

Another stratified analysis revealed that the materials FU (*p*<0.05), CL (*p*<0.0005), and LU (*p*<0.05) were significantly affected by storage (stratified UNI-ANOVA 2) (Table 3).

Although the factor location overall had no significant influence on bond strength, a stratified analysis revealed a significant interaction between “location” and “luting material” in groups FU (*p*<0.05), CL (*p*<0.0005), GR (*p*<0.0005), and RX (*p*<0.0005; stratified UNIANOVA 3) (Table 3). All mentioned groups revealed decreasing or stable (FU) tendencies from coronal to apical root parts, while RX revealed a significant increase in bond strength from coronal to middle/apical areas (*p*<0.0005; Tukey) (Table 3).

Failure modes were significantly affected by luting material (*p*<0.0005) (Table 4) and storage (*p*<0.0005, chi-square test). In group LU, the majority of samples revealed mixed failure initially and after storage cohesive failure increased slightly. Group RX revealed an equal distribution of failure between adhesive and dentin, post and luting material, and mixed failure, which drifted to a majority of failure between post and composite after storage.

The occurrence of voids inside the luting resin was significantly affected by luting material and location (*p*<0.0005, chi-square test) (Table 5). All materials revealed an occurrence of smaller and larger voids in over 90% of the samples.

**Fig. 2:**
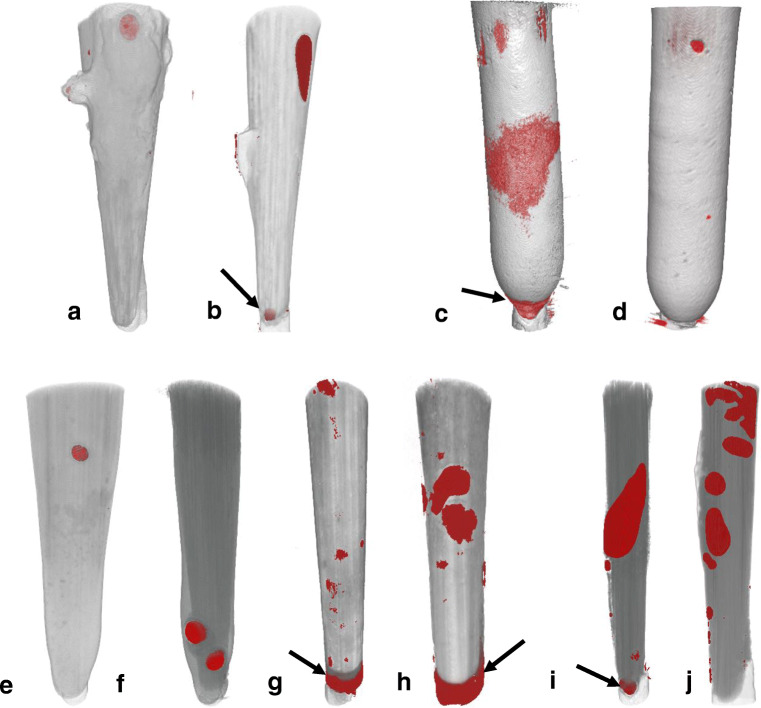
μCT images of two samples for each luting system (**a**–**j**): FU (a,b), CL (c,d), GR (e,f), LU (g,h), RX (i,j). Voids are depicted as red areas, while black arrows additionally mark voids between post and gutta-percha

The volumes acquired with μCT (Fig. 2) revealed the presence of voids in every sample of all groups varying only in size and location. In group GR as well as in group LU, the size and pattern of voids were comparable among the two samples tested in each group. One GR sample revealed a middle to large void, while the other contained two larger voids in the apical region (red). Both LU samples contained many large and smaller voids and one large void between post and gutta-percha each. In group FU, both samples contained one larger void each and a few smaller voids, while the second sample incorporated a void between post and gutta-percha. In group CL, the first sample revealed one large void and a void between post and gutta-percha as well as several large voids in the coronal area, while the second one revealed only small voids. In group RX, the first sample incorporated a very large void as well as few smaller voids, while the second one contained several larger ones which are distributed from the coronal to the apical area. In this group, both samples revealed a void between post and gutta-percha.

**Fig. 3:**
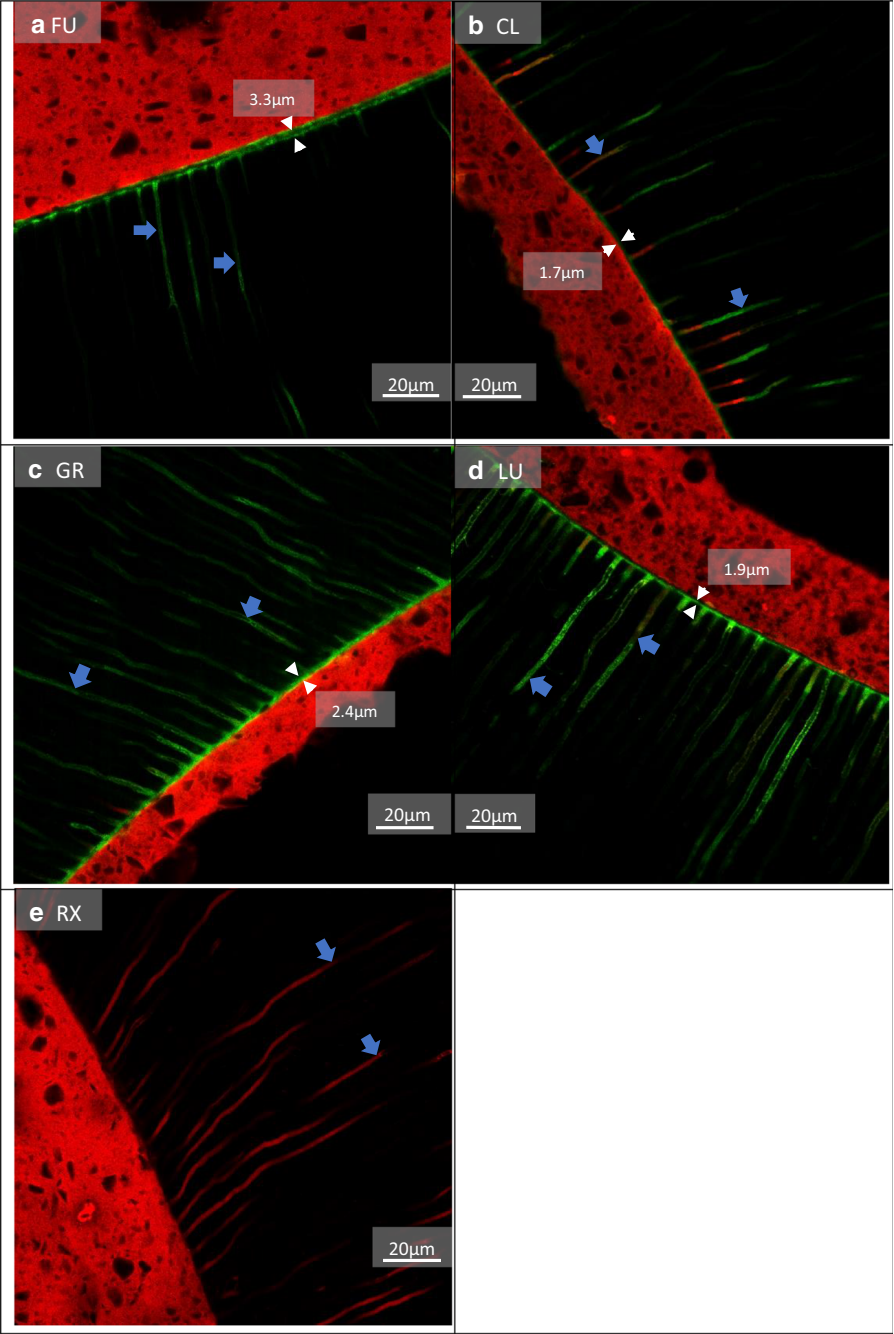
Fluorescence CLSM pictures, PT1: Ethanol (99%). **a** A very thick hybrid layer (3.3 μm) (white arrows) with a few long adhesive tags (blue arrows), that reach deep into the dentinal tubules. **b** A thin hybrid layer (1.7 μm) (white arrows) with fewer thin and long tags of adhesive and composite that reach deep into the dentinal tubules (blue arrows). **c** A clearly visible hybrid layer (2.4 μm) (white arrows) with mostly long adhesive tags (blue arrows). **d** A clearly visible hybrid layer (1.9μm) (white arrows) with a few thick and long, but mostly shorter adhesive tags that were partially infiltrated by composite (blue arrows). **e** No hybrid layer and fewer thin and long tags that reach deep into the dentinal tubules (blue arrows). Green = adhesive; red = composite/resin cement

**Fig. 4:**
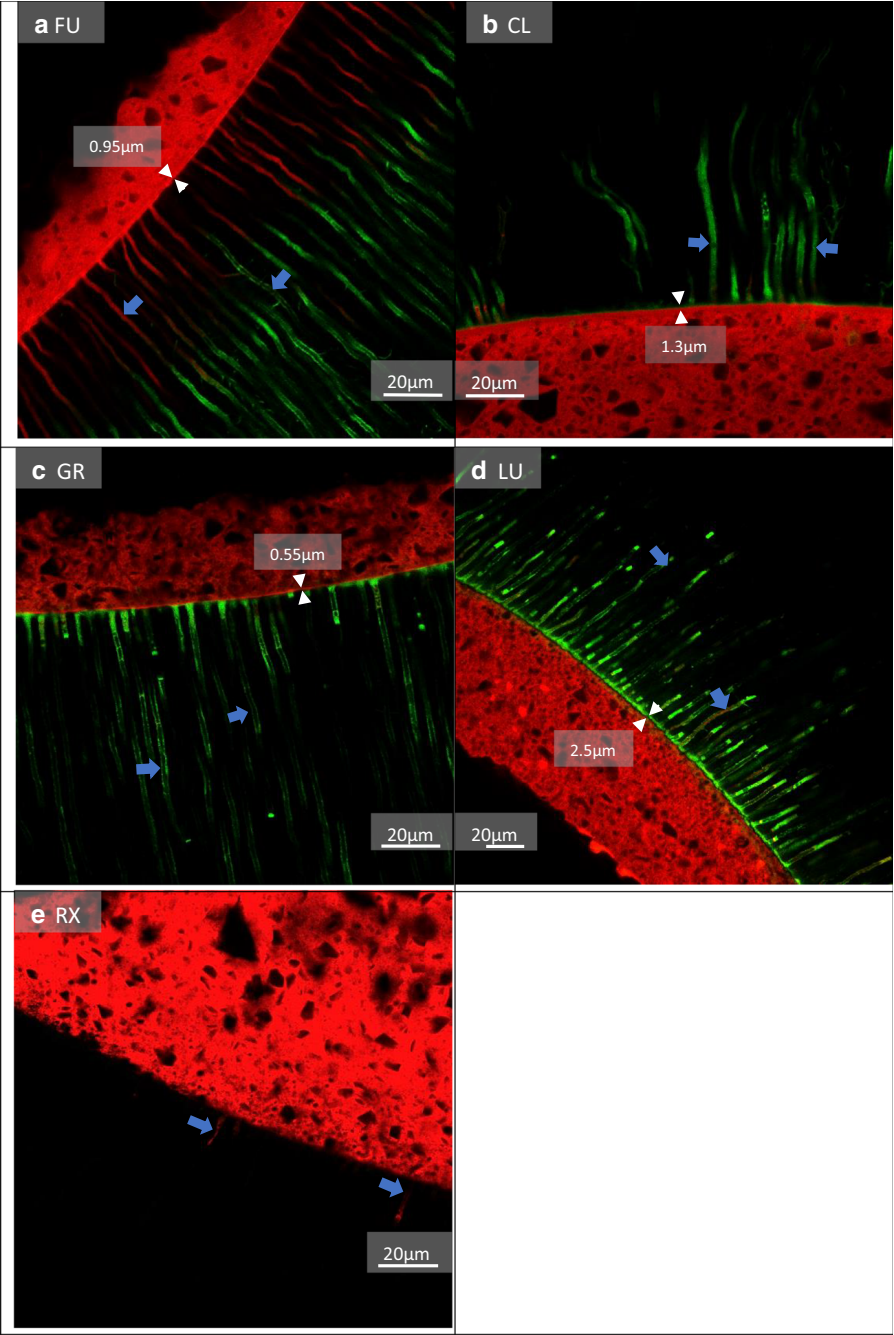
Fluorescence CLSM pictures, PT2: AH Plus Cleaner. **a** A very thin hybrid layer (0.95 μm) (white arrows) with many thin long composite and adhesive tags (blue arrows). **b** A thin hybrid layer (1.3 μm) (white arrows) with only a few longer adhesive tags positioned in bundles close to each other (blue arrows). **c** A very thin hybrid layer (0.55 μm) (white arrows) with long and short adhesive tags (blue arrows). **d** A thick and clearly visible hybrid layer (2.5 μm) (white arrows) with many longer adhesive tags and little composite infiltrations (blue arrows). **e** No hybrid layer and two short tags (blue arrows). Green = adhesive; red = composite/resin cement

**Fig. 5:**
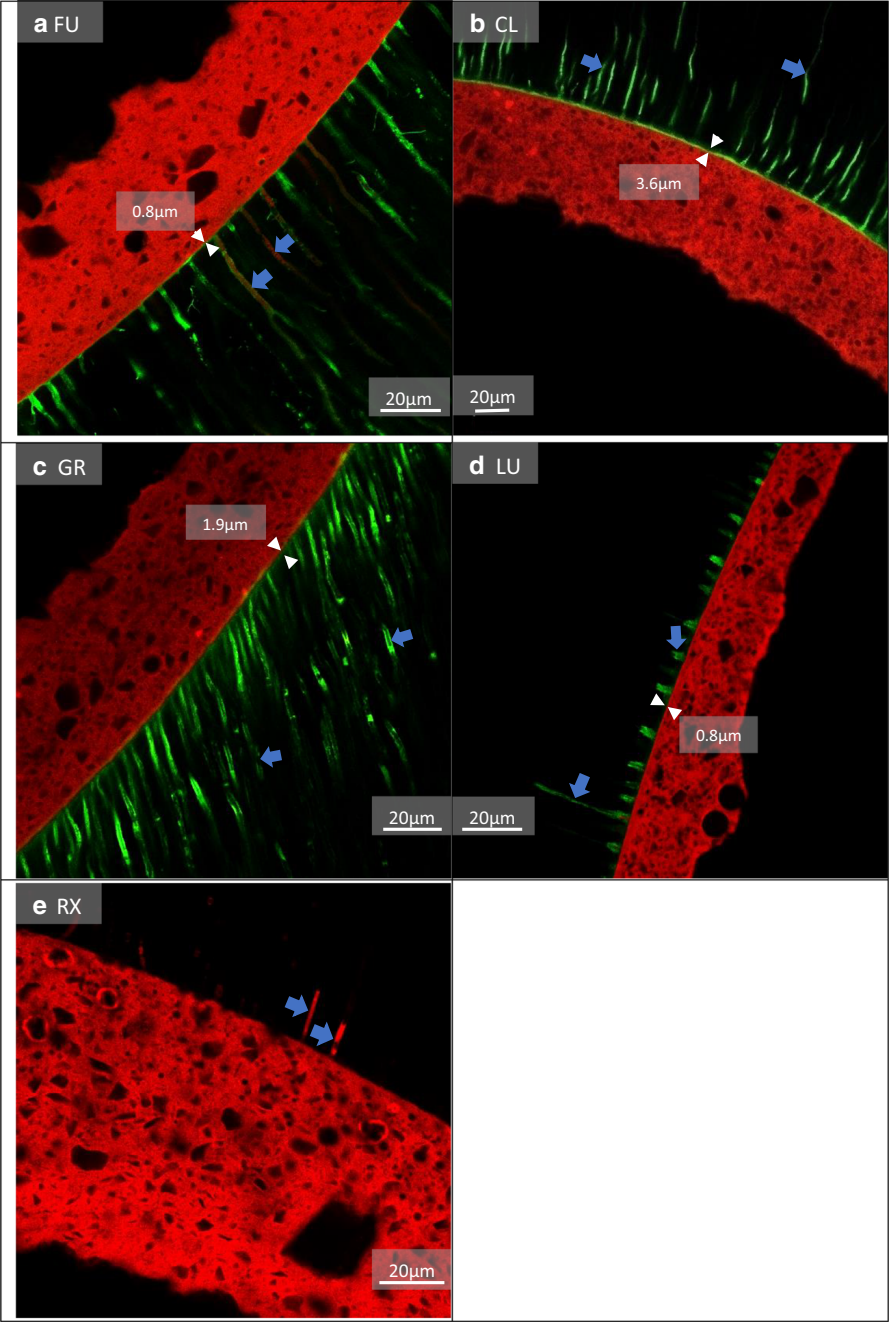
Fluorescence CLSM pictures, PT3: control, distilled water. **a** A very thin hybrid layer (0.8 μm) (white arrows) with many longer and shorter adhesive tags (blue arrows). **b** A thick hybrid layer (3.6 μm) (white arrows) with many longer adhesive tags (blue arrows). **c** A clearly visible hybrid layer (1.9 μm) (white arrows) with a few long and many shorter adhesive tags (blue arrows). **d** A very thin hybrid layer (0.8 μm) (white arrows) with very short adhesive tags (blue arrows). **e** No hybrid layer and few shorter tags (blue arrows). Green = adhesive; red = composite/resin cement

The CLSM images revealed a prominent hybrid layer in groups FU, CL, GR, and LU, while group RX showed no hybrid layer. In some groups, the infiltration depths and thickness of adhesive tags differed depending on the pre-treatment used (Figs. 3, 4 and 5). LU revealed a thicker hybrid layer with PT1 compared to PT3. RelyX Unicem 2 revealed more and longer resin tags with PT1 that reached deeper into the dentinal tubules compared to those with PT2 and PT3.

## Discussion

The present study shows that pre-treatment, storage, and luting materials significantly affected bond strength. Interactions between the factors pre-treatment and luting material, storage, and luting material as well as location and luting material could be observed.

Aging of the adhesive–dentin interface is a synergistic process caused by the degradation of each component [22]. Degradation of collagen fibrils and a leakage of adhesive from the hybrid layer are two important factors during this process [23]. An entrapment of water inside the adhesive resin is supposed to be one of the main causes for collagen and subsequently occurring hybrid layer degradation [22, 24]. Ethanol is able to facilitate water evaporation from dentin, therefore reducing its intrinsic wetness [9]. In our study, rinsing of the root canal using ethanol had a positive effect on the bond strength of one SE resp. universal adhesive system (LuxaBond Universal) and the investigated SAR cement (RelyX Unicem 2). In combination with the ethanol pre-treatment, LuxaBond Universal reached the highest bond strength among all groups (Table 3). CLSM analyses of LuxaBond Universal’s hybrid layer formation with ethanol pre-treatment (Fig. 3) (approximately 3.3 μm) revealed an improved adhesive infiltration compared to the control group (Fig. 5) (approximately 1.9 μm) using distilled water. Similar results were found in the RelyX Unicem 2 group, where the infiltration depth of the SAR cement was improved using ethanol. Previous research [20] corroborated the present data demonstrating beneficial effects of ethanol pre-treatment on RelyX Unicem 2 in the apical parts of the root canal. The same effect was also observed on Futurabond U (in self-etch mode) [20], another universal adhesive, which does not align with the results of our study. The effects of ethanol pre-treatment on bond strength have been shown to be highly dependent on the luting material [9]. Although ethanol irrigation did not improve bond strengths in groups Futurabond U, Clearfil DC Bond and Gradia Core SE Bond, it also did not hamper bonding, which corresponds to results by Bronzato et. al [25]. Therefore, it can be speculated that other factors overshadowed the pre-treatment’s effect. Similar to our study design, Cecchin et al. [13] applied ethanol for 60 s onto root canal dentin and did not achieve improved bond strengths. The authors argued that residual water might take longer to be evaporated completely. Thus, an application of ethanol longer than 60 s might increase bond strengths of these adhesives, as well. Futurabond U was not light-cured before the application of Rebilda DC; Gradia Core SE Bond was rubbed into the root canal walls very shortly, both according to the manufacturer’s instructions (Table 1). Since active application of SE and universal adhesives into root canal dentin proved to increase bond strength [19, 26], a very short active application could hamper the effect of ethanol pre-treatment on bond strength. Tezvergil-Mutluay et al. [27] observed that tert-butanol, which is included in AH Plus Cleaner (PT2), is able to deactivate matrix metalloproteases (MMPs) inside human dentin. These MMPs can be released and activated during dentin bonding leading to a thinning and disappearance of collagen fibrils in aged dentin [28] which has been confirmed to occur in vivo at the deepest part of the hybrid layer [29]. However, MMPs inside the collagen matrix can only be activated if enough collagen fibers are exposed. Since SE adhesives do not expose collagen and the attached MMPs to the same extent as ER adhesives, they are less likely to activate MMPs [30]. We observed no significant difference in PT2’s bond strength compared to ethanol (PT1) or distilled water (PT3). Therefore, it can be concluded that an overall possible positive effect of tert-butanol pre-treatment on bond strength durability could not be shown in the present study set-up. Nonetheless, Gradia Core SE Bond samples treated with AH Plus Cleaner revealed an increase in bond strength after TC and storage (Table 3).

The push-out tests revealed higher mean bond strengths for one universal adhesive system (LuxaBond Universal) compared to the other tested systems, including an often-investigated SAR cement (RelyX Unicem 2). Previous research showed significantly higher bond strength values for posts luted with SAR cements compared to the application of ER or SE adhesives in combination with core build-up materials [14]. According to Wang et al., SE adhesives contain different types and concentrations of functional monomers that interact variously with dental hard tissue resulting in adhesive interfaces with different characteristics [31]. Universal adhesives chemically bond to dental hard tissue via functional monomers such as 10-methacryloyloxydecyl dihydrogen phosphate (10-MDP) which forms insoluble calcium salts with hydroxylapatite resulting in stable bonds [32, 33]. A very recent study revealed that the self-assembled nanolayering effect of 10-MDP contributed to the mechanical properties of an adhesive, possibly increasing its bonding durability to dentin [34]. According to the manufacturer’s information (Table 2) Clearfil DC Bond contains MDP monomers, but revealed a significant decrease in bond strength after TC and storage (Table 3). Futurabond U, LuxaBond Universal, Gradia Core SE Bond, and RelyX Unicem 2 also contain adhesion-promoting functional monomers, albeit not all are named specifically by their manufacturers (Table 2). LuxaBond Universal’s bond strength to root dentin might have benefited more from the adhesive interface formed by its functional monomers compared to the other luting agents. The two investigated universal adhesives, LuxaBond Universal and Futurabond U, as well as the SE adhesive Clearfil DC Bond all contain 2-Hydroxyethyl methacrylate (HEMA) (Table 2), a functional monomer which is able to facilitate adhesion to wetted surfaces, because of its hydrophilic nature [18]. It was shown that an adequate concentration of HEMA improves monomer diffusion, infiltration, and hybrid layer formation resulting in increased immediate bond strengths [35]. The CLSM images (Figs. 3, 4, and 5) of this study partially support these findings as they revealed a prominent hybrid layer for all adhesives containing HEMA. A disadvantage when including this monomer is its affinity to water sorption [18], because it contributes to a degradation of the hybrid layer [36] and higher concentrations might decrease the adhesive’s mechanical properties [18].

**Table 2:** Composition of all five luting systems according to manufacturers’ information

**Futurabond U**	**Clearfil DC Bond**	**Gradia Core SE Bond**	**LuxaBond Universal**	**RelyX Unicem 2**
*Liquid I:* bisphenol-a-(di)-methacrylate (bis-GMA), 2-hydroxyethyl methacrylate (HEMA), hydroxyl-ethyl-di-methacrylate (HEDMA), sour adhesive monomers, urethane-di-methacrylate (UDMA) catalyst. *Liquid II:* catalyst and initiator solvent: ethanol	*Liquid A:* HEMA, bis-GMA, 10-Methacryloyl oxydecyl dihydrogen phosphate (10-MDP), DL-campherchinon, benzoyl peroxide, polyethylene glycol, silanized silica filler *Liquid B:* solvent: water and ethanol	*Liquid A:* 2-hydroxy-1,3-dimethacryloxypropane, 2-2‘-ethylendidioxi-diethyldimethacrylate, 7,7,9 (or 7,9,9,) -trimethyl-4,13-dioxo-3,14-dioxa-5,12-diazahexadecane-1,16-diylbismethacrylate solvent: ethanol *Liquid B:* solvent: ethanol & 2-2‘-[(4-methylphenyl) imino] bis-ethanol	*Bond A:* HEMA, bis-GMA based hydrophilic and hydrophobic and sour resin matrix, di-benzoyl peroxide/ benzoyl peroxide and additives *Bond B:* starter solvent: ethanol and water	*Base:* methacrylates with phosphor acid groups, other methacrylates silanized fillers, initiators stabilisators, rheological additives *Catalyst:* methacrylates, alkaline fillers, initiators, stabilisators, pigments, rheological additives
**Rebilda DC**	**Clearfil DC Core**	**Gradia Core**	**LuxaCore Z Dual**	
*Base:* UDMA, 1,10-decandioldimethacrylate (DDDMA), bis-GMA *Catalyst:* UDMA, DDDMA, bis-GMA, benzoyl peroxide	*Paste A:* bis-GMA, hydrophobic/hydrophilic aliphatic/aromatic dimethacrylate, DL-campherchinon, initiators, and fillers *Paste B:* tri-ethylene-glycol-di-methacrylate (TEGDMA), other di-methacrylates and accelerators	*Base:* 7,7,9 (or 7,9,9,) -trimethyl-4,13-dioxo-3,14-dioxa-5,12-diazahexadecane-1,16-diylbismethacrylate, 2-2‘-ethylendidioxi-diethyldimethacrylate, 2,2- dimethyl-1,3-propanediylbismethacrylate *Catalyst:* 7,7,9 (or 7,9,9,) -trimethyl-4,13-dioxo-3,14-dioxa-5,12-diazahexadecane-1,16-diylbismethacrylate, 2-hydroxy-1,3-dimethacryloxypropane, di-benzoyl-peroxide, 1,6-di-tert-butyl-p-cresole	di-methacrylate, barium glass, pyrogenic silica, nano filler zirconia dioxide inside a bis-GMA based matrix

**Table 3:** Mean bond strength values (MPa) plus standard deviation of the five post and core systems divided by pre-treatment, location, and storage

Adhesives	PT	Region inside the root canal								
		Coronal mean (SD)		Middle mean (SD)		Apical mean (SD)		Ʃ mean (SD)		
		Initial	TC	Initial	TC	Initial	TC	Initial	TC	Ʃ
1. FU	Ethanol	15.6 (7.4)	11.8 (5.3)	12.9 (6.5)	10.7 (4.8)	15.5 (9.9)	12.9 (5.8)	**14.7** **(7.9)**	**11.8** **(5.2)**	**13.2** **(6.8)**
	AH^+^- Cleaner	17.1 (4.8)	13.7 (4.8)	13.6 (6.7)	11.9 (5.2)	16.2 (10.0)	11.8 (6.3)	**15.6** **(7.4)**	**12.5** **(5.4)**	**14.0** **(6.6)**
	Control	15.2 (7.3)	11.9 (2.8)	12,85 (5.7)	11.1 (4.6)	14.8 (4.5)	17.5 (4.1)	**14.3** **(5.9)**	**13.5** **(4.7)**	**13.9** **(5.3)**
	**Ʃ**	**15.97** **(6.5)**	**12.5** **(4.4)**	**13.1** **(6.1)**	**11.2** **(4.8)**	**15.5** **(8.3)**	**14.0** **(5.9)**	**14.9** **(7.1)**	**12.6** **(5.1)**	
	**Ʃ**	**14.2** **(5.7)**		**12.2** **(5.5)**		**14.7** **(7.2)**				
2. CL	Ethanol	20.2 (2.9)	13.9 (3.4)	17.6 (2.7)	14.8 (4.8)	13.9 (3.5)	9.7 (2.2)	**17.2** **(4.0)**	**12.8** **(4.2)**	**15.0** **(4.6)**
	AH^+^- Cleaner	16.1 (3.2)	13.3 (4.5)	15.99 (3.3)	11.98 (3.0)	14.0 (5.0)	5.9 (3.9)	**15.4** **(4.0)**	**10.4** **(5.0)**	**12.8** **(5.1)**
	Control	16.9 (5.1)	14.6 (5.1)	14.8 (4.3)	12.9 (4.7)	13.8 (7.4)	10.7 (5.5)	**15.2** **(5.7)**	**12.7** **(5.2)**	**13.95** **(5.6)**
	**Ʃ**	**17.7** **(4.2)**	**13.9** **(4.3)**	**16.1** **(3.6)**	**13.2** **(4.3)**	**13.9** **(5.4)**	**8.8** **(4.5)**	**15.9** **(4.7)**	**11.97** **(4.9)**	
	**Ʃ**	**15.8** **(4.6)**		**14.7** **(4.2)**		**11.3** **(5.8)**				
3. GR	Ethanol	17.9 (5.9)	16.7 (5.1)	13.7 (3.5)	13.0 (2.1)	12.7 (4.9)	12.7 (5.3)	**14.7** **(5.2)**	**14.1** **(4.6)**	**14.4** **(4.9)**
	AH^+^- Cleaner	17.0 (3.6)	20.7 (4.8)	13.3 (2.3)	13.0 (2.6)	13.95 (2.9)	16.9 (4.9)	**14.7** **(3.3)**	**16.9** **(5.2)**	**15.8** **(4.4)**
	Control	16.9 (5.6)	17.4 (5.1)	15.6 (4.8)	13.1 (2.0)	12.7 (2.8)	15.3 (2.1)	**15.1** **(4.8)**	**15.3** **(3.7)**	**15.2** **(4.3)**
	**Ʃ**	**17.3** **(5.0)**	**18.2** **(5.2)**	**14.2** **(3.7)**	**13.0** **(2.2)**	**13.1** **(3.6)**	**14.98** **(4.5)**	**14.8** **(4.5)**	**15.4** **(4.6)**	
	**Ʃ**	**17.7** **(5.1)**		**13.6** **(3.1)**		**14.0** **(4.2)**				
4. LU	Ethanol	23.8 (8.1)	19.7 (5.6)	25.7 (9.7)	23.1 (9.9)	21.2 (5.4)	18.6 (7.6)	**23.6** **(7.9)**	**20.5** **(7.9)**	**22.0** **(8.0)**
	AH^+^- Cleaner	20.3 (9.2)	16.2 (6.0)	18.8 (7.5)	18.5 (8.1)	21.2 (7.6)	21.3 (7.9)	**20.1** **(8.0)**	**18.6** **(7.5)**	**19.4** **(7.7)**
	Control	18.7 (7.6)	15.4 (5.7)	15.3 (7.5)	14.4 (7.1)	14.1 (6.5)	11.7 (5.8)	**16.0** **(7.3)**	**13.8** **(6.2)**	**14.9** **(6.8)**
	**Ʃ**	**20.9** **(8.4)**	**17.1** **(5.9)**	**19.9** **(9.2)**	**18.7** **(9.0)**	**18.8** **(7.2)**	**17.2** **(8.1)**	**19.9** **(8.3)**	**17.6** **(7.7)**	
	**Ʃ**	**19.0** **(7.5)**		**19.3** **(9.0)**		**18.0** **(7.7)**				
5. RX	Ethanol	16.6 (3.5)	14.2 (3.6)	18.7 (5.2)	20.1 (8.5)	19.5 (5.2)	19.1 (4.4)	**18.2** **(4.7)**	**17.8** **(6.3)**	**18.0** **(5.5)**
	AH^+^- Cleaner	11.9 (6.7)	9.8 (3.7)	19.7 (7.7)	19.1 (7.2)	19.6 (6.8)	13.9 (4.0)	**17.0** **(7.8)**	**14.3** **(6.4)**	**15.7** **(7.2)**
	Control	11.8 (6.3)	11.4 (5.8)	15.1 (4.3)	16.4 (5.0)	17.2 (6.9)	15.5 (5.8)	**14.7** **(6.2)**	**14.4** **(5.8)**	**14.6** **(6.0)**
	**Ʃ**	**13.5** **(6.0)**	**11.8** **(4.7)**	**17.8** **(6.1)**	**18.5** **(7.0)**	**18.7** **(6.3)**	**16.2** **(5.1)**	**16.7** **(6.5)**	**15.5** **(6.3)**	
	**Ʃ**	**12.6** **(5.4)**		**18.2** **(6.5)**		**17.5** **(5.8)**				

All results in form of sums were highlighted in bold

LuxaBond Universal was the only adhesive applied in two coats possibly improving its immediate dentin bond strength as research suggests that multiple coats of universal adhesives resulted in higher immediate bond strengths to dentin [37]. Adhesive layering may improve etching and a thicker adhesive layer should increase bond strength against stress concentrations [38]. This could further explain how LuxaBond Universal maintained its high bond strengths in the coronal, middle, and apical parts of the root canal.

As studies have shown that immediate dentin bond strength can differ significantly from long-term bond strength values [39], we decided to include aging and storage of samples. Futurabond U, Clearfil DC Bond, and LuxaBond Universal showed a decrease in bond strength after TC and storage which correlated to a change in failure modes. Futurabond U and Clearfil DC Bond revealed more failures between post and luting cement after storage indicating a change in the weak link of this interface (Table 4). Post cement bonding depends on micro-mechanical interlocking affected by post-surface topography as well as chemical bonding [40]. A silanization was recommended by the manufacturers for all posts except for LuxaPost (pre-silanized) and RelyX Fiber Post which relies on micromechanical interlocking. Silanization is supposed to create a chemical interaction between organic matrices on one hand and inorganic structures, e.g. glass fibers inside fiber posts, on the other [41]. If and how the effect of silanization improves bond strength of fiber posts is still a controversial topic [42]. Benefits of silane application are mostly attributed to a higher wettability of silanized post surfaces and depend on the type of post and luting agent [40]. An incomplete chemical bond could lead to gap formation at the post surface and easier infiltration of water during storage resulting in increased failure and decreased bond strength at the post surface. LuxaBond Universal on the other hand revealed six times more cohesive failure inside the post after TC and storage (Table 4) possibly caused by a very stable bond between adhesive and dentin as well as post and cement. Moreover, research indicates that TC and water storage resulted in a decreased fracture load of fiber posts [43]. Although hydrolytic degradation is considered to be the main mechanism in decreasing bond strength over time [22], the experimental set-up of our study has to be considered a worst-case scenario, because clinically, it is very unlikely that every bonding surface of the post and core build-up will be directly exposed to water

**Table 4:** Crosstab comparing the five different post and core systems before and after TC and storage based on their failure modes after push-out (pooled data for location)

	Failure modes after push-out (initial and stored)							
	Adhesive dentin		Post and composite		Cohesive post		Mixed failure	
Adhesives	Initial	Stored	Initial	Stored	Initial	Stored	Initial	Stored
1. FU	30.1%	23.1%	8.8%	16.2%	0	0	11.1%	10.6%
2. CL	3.7%	5.6%	32.9%	36.1%	0.9%	0	12.5%	8.3%
3. GR	14.4%	9.3%	27.8%	33.3%	0	0	7.9%	7.4%
4. LU	15.7%	13.4%	12.5%	11.1%	1.9%	11.1%	19.9%	14.4%
5. RX	17.1%	19.9%	13.4%	17.6%	0	0	19.4%	12.5%

The interaction between location and the luting materials Futurabond U, Clearfil DC Bond, and Gradia Core SE Bond revealed an overall decreasing trend in bond strength from the coronal to the apical region. It was proven that dentinal tubules decrease in size and number from the cervical to the apical area [44] possibly compromising bonding. An entrapment of solvent inside the hybrid layer caused by incomplete solvent evaporation [46] could have also caused the decrease in bond strength. Although all adhesives of this study have been dried with air, some have been air-dried longer than others (Table 1). Moreover, it was proven that thorough air drying is not effective enough to remove all excess solvent [47]. All adhesives used in this study were dual-curing and Futurabond U was the only one used without additional light polymerisation, as instructed by the manufacturer. Therefore, it could be assumed that complete polymerisation of this adhesive in the apical parts of the post space might be impeded or solely relys on the chemical curing mode. RelyX Unicem 2 revealed lower coronal bond strengths that increased in the middle and apical thirds (Table 3). Jha and Jha  [48] found similar results for RelyX cement inside the root canal and argued that the narrow space of the apical post space leads to a thinner cement layer compared to that of the coronal region resulting in less polymerizing shrinkage and higher bond strength.

In the present study, the void formation was significantly affected by the luting material. The number of voids was comparable among groups, but the size of voids differed. One possible explanation could be related to the flow properties of each material which depend on their composition. The composites Rebilda DC, Clearfil DC Core Plus, and LuxaCore Z dual contained di-methacrylates, such as UDMA, TEGDMA, and Bis-GMA, among other substances (Table 2). The amount of TEGDMA, which serves as a diluent, was proven to be inversely correlated to the viscosity of an UDMA mixture  [49]. Gradia Core had to be applied with an application gun provided by the manufacturer, since it only flows under pressure due to its high filler content (75%) and thixotropic character [50]. In the current study, most voids, especially the larger ones, occurred at the apical third of the root which is in concordance with results by Chang et al. [51]. The authors explained that apical parts of the post space are narrow and harder to clean which could foster void formation by impeding proper adaption of the adhesive. The occurrence of voids inside the resin in this study was observed using a digital microscope (Table 5) as well as a laboratory μCT (Fig. 2). Note that the μCT samples solely analyzed two samples per group exemplary. However, both methods revealed concordant results in the number and sizes of entrapped voids. Although it is very time-consuming to produce μCT images, a clear advantage of this technique is its non-destructive or non-invasive nature [52]. The highlight of voids within the cement layer was achieved by separating different phases of restoration based on their density (segmentation).

**Table 5:** Crosstab comparing the five different post and core systems based on the quantity and size of voids in the luting material

Voids	Post- and core systems				
	1. FU	2. CL	3. GR	4. LU	5. RX
Void-free	9.3%	7.9%	8.3%	4.6%	4.2%
Small voids	82.9%	79.2%	76.4%	72.7%	70.4%
Bigger/numerous voids	7.9%	13.0%	15.3%	22.7%	25.5%

Voids and inhomogeneities can occur due to mixing or during application of the resin/cement into the root canal [53] as a result of air entrapment [54]. There is no consensus among researchers regarding the influence of voids inside post and core build-ups. Uzun et al. found no correlation between bond strength and occurrence of voids [55]. Others concluded that the presence of voids is disadvantageous, since they change stress concentrations on the surface between dentin and resin which leads to reduced bond strengths of fiber posts [53]. However, Lorenzoni et al. [54] speculated that these voids could serve as free surfaces for resin cement contraction and could therefore lead to stress release during polymerization shrinkage which could improve bond strength. This could explain why LuxaCore Z dual revealed higher bond strengths despite larger void formation. Note, however, that Clearfil DC Core Plus as well as LuxaCore Z dual also revealed large voids in the apical area directly below the post or around the post tip (Fig. 2; c,d and g,h). According to Silva et al. [53], this phenomenon is caused by air entrapment in the confined apical post space leading to a larger distance between post and gutta-percha and potentially resulting in unfavourable outcomes [56].

## Conclusion

The current study revealed that SE dual-curing adhesives are equally reliable for post cementation as SAR cement. While the universal adhesive LuxaBond Universal revealed higher bond strength than RelyX Unicem 2, both benefitted from root canal pre-treatment using ethanol. The immediate- and long-term bond strengths were increased and remained stable, while penetration into the dentinal tubules was facilitated. LuxaBond Universal revealed the highest immediate bond strength values which remained stable from the coronal to the apical region. It is apparent that the materials reacted differently to water exposure during TC and storage. This suggests that hydrolytic degradation decreased bonding performances of only some materials. The void formation was observed in all materials despite a standardized application method; its clinical implication must be investigated further.

## Declarations

### Disclaimer

The funders had no role in design, conduct, evaluation, or interpretation of the study, or writing the paper.
